# Foliar Application of CeO_2_ Nanoparticles Alters Generative Components Fitness and Seed Productivity in Bean Crop (*Phaseolus vulgaris* L.)

**DOI:** 10.3390/nano11040862

**Published:** 2021-03-28

**Authors:** Hajar Salehi, Abdolkarim Chehregani Rad, Ali Raza, Jen-Tsung Chen

**Affiliations:** 1 Laboratory of Plant Cell Biology, Department of Biology, Bu Ali Sina University, Hamedan 65178-38695, Iran; hajarsalehi@ymail.com; 2 Key Lab of Biology and Genetic Improvement of Oil Crops, Oil Crops Research Institute, Chinese Academy of Agricultural Sciences (CAAS), Wuhan 430062, China; alirazamughal143@gmail.com; 3 Department of Life Sciences, National University of Kaohsiung, Kaohsiung 811, Taiwan

**Keywords:** cerium dioxide nanoparticles, reproductive phase, pollen viability, seed productivity, food safety

## Abstract

In the era of technology, nanotechnology has been introduced as a new window for agriculture. However, no attention has been paid to the effect of cerium dioxide nanoparticles (nCeO_2_) on the reproductive stage of plant development to evaluate their toxicity and safety. To address this important topic, bean plants (*Phaseolus vulgaris* L.) treated aerially with nCeO_2_ suspension at 250–2000 mg L^−1^ were cultivated until flowering and seed production in the greenhouse condition. Microscopy analysis was carried out on sectioned anthers and ovules at different developmental stages. The pollen’s mother cell development in nCeO_2_ treatments was normal at early stages, the same as control plants. However, the results indicated that pollen grains underwent serious structural damages, including chromosome separation abnormality at anaphase I, pollen wall defect, and pollen grain malformations in nCeO_2_-treated plants at the highest concentration, which resulted in pollen abortion and yield losses. On the ovule side, the progression of development only at the highest concentration was modified in the two-nucleated embryo sac stage, probably due to apoptosis in nuclei. Nevertheless, the findings confirmed the more pronounced vulnerability of male reproductive development under nCeO_2_ exposure than female development. The higher concentration decreased seed productivity, including seed set in either pods or whole plant (13% and 18% compared to control, respectively). The data suggested the potential application of nCeO_2_ at optimal dosages as a plant productivity ameliorative. However, a higher dosage is considered as an eco-environmental hazard. To our best knowledge, this is the first study analyzing reproductive plant response upon exposure to nCeO_2_.

## 1. Introduction

In the past decade, applied nanotechnology has entered into the agricultural-associated sectors industrially and commercially. The widespread application of nanoparticles (NPs) inevitably led to appearances of NPs in various edible plants. Therefore, due to the significant importance of NPs fate of entering into plants, extensive studies have concerned reciprocal NPs–plants interaction at the physio-chemical level [1,2,3]. Nevertheless, extra information is needed to reach relatively agreements on the safety of NPs environmentally before their use worldwide. Plant reproduction and productivity as a final and critical development stage have been rarely studied in the literature reviews. This could be due to less endeavor of researchers in doing long-term experiments. Indeed, the difficulty of investigating the internal processes of plant development (i.e., gametogenesis and sporogenesis) that occur inside the genital organs of the flower (stamen and ovary) is another obstacle to this research.

Plant growth and development can be influenced by both biotic and abiotic stresses at any time of the plant life cycle [4,5]. However, the intensity and nature of detriments, the recovery possibilities, yield, and productivity depend on the developmental stage at which a plant faces an unfavorable conditions [6]. For instance, the sensitivity to drought and high-temperature stress is remarkably mortal during the reproductive phase due to the vulnerability of the involved processes, especially meiosis of microspore and megaspore mother cells in male and female organs, respectively, and also pollen development [7,8].

Currently, cerium oxide NPs (nCeO_2_) (a type of lanthanide metal oxide) have been used in multiple applications among other engineered NPs due to their unique redox, surface area, and catalytic features [9]. These NPs have been demonstrated to show variable behavior when absorbed by plants. Several factors such as plant species type, media condition, and exposure time are critical in nCeO_2_ behavior besides their characteristics, including size, physicochemical properties, and amount [2,10,11,12]. For instance, lower biomass, reduced pigments such as chlorophyll, increased antioxidant enzymes including catalase, peroxidase, and superoxide dismutase resulted from high concentrations of nCeO_2_ through foliar and irrigation applications [2,13]. Moreover, the inhibition of enzyme activities under severe conditions has also been documented [2]. However, lower concentrations represent the higher antioxidant activity to scavenge the reactive oxygen species (ROS) [14,15], which result in the protection of cellular processes such as photosynthesis against oxidative stresses and finally promote stress tolerance and plant growth [11,16,17]. Our recent study of applied nCeO_2_ on solid agar medium provided promising evidence of their role in adjustments of secondary metabolism and the ionomic balance of *Phaseolus vulgaris* L. [3].

The literature suggests that nCeO_2_ has considerable effects on the vegetative stages of plant growth and development, showing physiological, biochemical, and molecular statues of vegetative cells. However, to adequately assess their mechanistic understanding of plant productivity’s determinative phase (i.e., flowering stage and seed production), the information is still lacking. Only a few studies have provided numerical measurements on the yield and nutritional status of edible parts of plants. For example, nCeO_2_ at 62.5 mg kg^−1^ produced a higher total number of tomatoes than 125 and 500 mg kg^−1^ concentrations [18]. In another study, two concentrations of nCeO_2_ (100 and 400 mg kg^−1^) caused a delay in flowering by one week and reduced the size of starch grains in wheat plants but did not result in final biomass and yield changes [13]. On the other hand, exposure of bean plants to nanoceria (62.5 and 125 mg kg^−1^) caused down-regulation of several proteins associated with nutrient storage like phaseolin and lectins [19].

Based on the available literature and followed by our published study [2], we came to investigate a life cycle of bean plants to partly uncover the existing knowledge gaps in understanding of the mechanisms underlying the nCeO_2_ effects, particularly on the hidden aspects of the yield and seed productivity. To address this important topic, we first monitored the gametogenesis and sporogenesis processes in both male and female organs (stamen and ovary) following by pollen grain, embryonic sac, and embryo development. Secondly, the total yield, protein content, and sodium dodecyl sulfate-polyacrylamide gel electrophoresis (SDS-PAGE) of seeds were also analyzed. Thus, the present study aimed to verify possible changes in reproductive pathways that may explain some of the reported contradictory findings regarding plant yield after exposure to nCeO_2_ treatment. It’s worth noticing that evaluation of growth stages of bean plants (before flowering) exposed to nCeO_2_ was addressed in our previous research [2], and the current work is a follow-up to previous findings.

## 2. Results and Discussion

**Figure 1:**
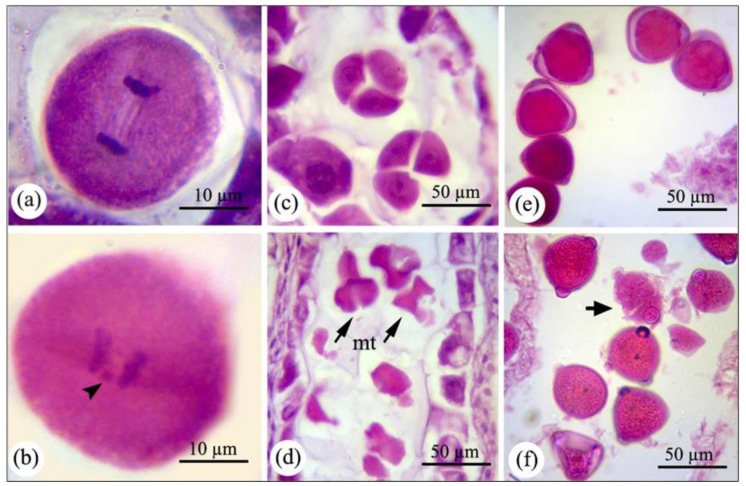
The microsporogenesis process occurred in control and 2000 mg L^−1^ nCeO_2_-treated plants. (**a**) Pollen mother cell at anaphase I in control plants (**b**) pollen mother cell at anaphase I, which chromosomes separation has not been appropriately accomplished and led to generating the laggard chromosome (arrowhead). (**c**) Pollen mother cell at the tetrad stage in control plants. (**d**) Abnormal and degenerated tetrads (arrows) in nCeO_2_-treated plants. (**e**) Mature pollen grains in control and treated plants. (**f**) Degenerated (arrow), and misshaped pollens are visible in anthers of plants exposed to higher dosages of nCeO_2_. mt, microspore tetrad.

**Figure 2:**
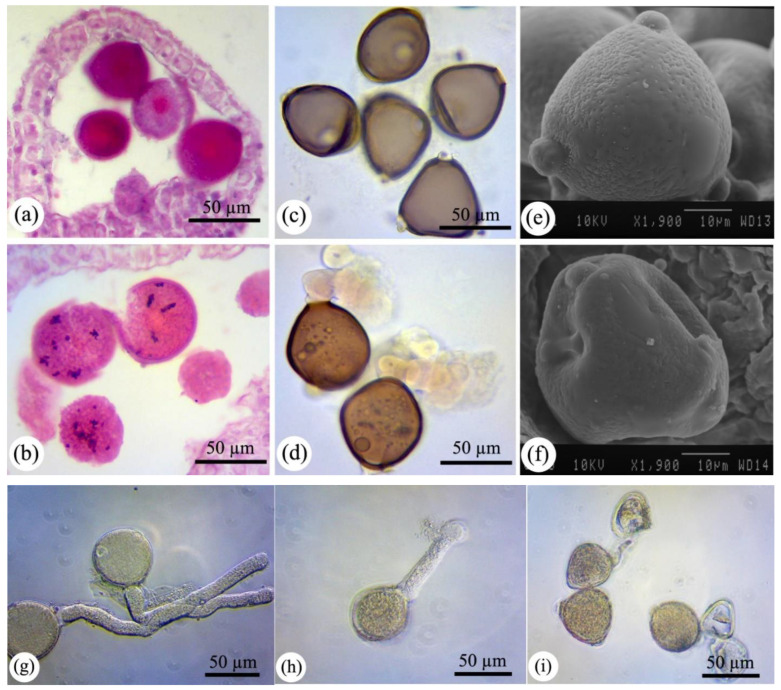
The development of pollen grains in control (**a**,**c**,**e**,**g**) and nCeO_2_-treated plants (**b**,**d**,**f**,**h**,**i**). (**b**) Shows pollen grains enriched by starch and some degenerated pollens in nCeO_2_-treated plants. Pollen grains stained with acetocarmine show cytoplasmic exudation especially upon exposure to the highest concentration (**d**). The scanning electron micrograph of pollen grains shows abnormal pollen folding in structure in plants treated with 2000 mg L^−1^ nCeO_2_ (**f**). The germination of pollen grains is significantly decreased in plants exposed to nCeO_2_ (**h**,**i**) compared to control (**g**).

**Figure 3:**
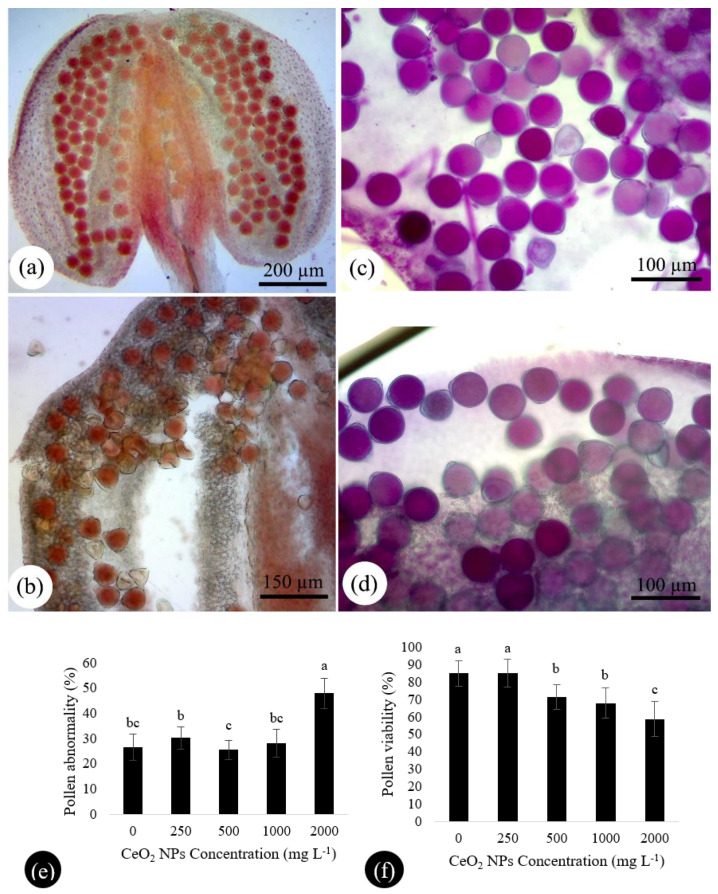
Acetocarmine (**a**,**b**) and Alexander (**c**,**d**) staining of pollens taken from mature anthers in control (**a**,**c**) and 2000 mg L^−1^ nCeO_2_ treatment (**b**,**d**). The anthers of control plants indicate higher viability by showing more colorful and complete filling pollens. In contrast, incomplete filing pollens with crumpled shapes indicate less viability. Pollen abnormality and pollen viability at different concentrations compared to control are represented in (**e**) and (**f**), respectively.

**Figure 4:**
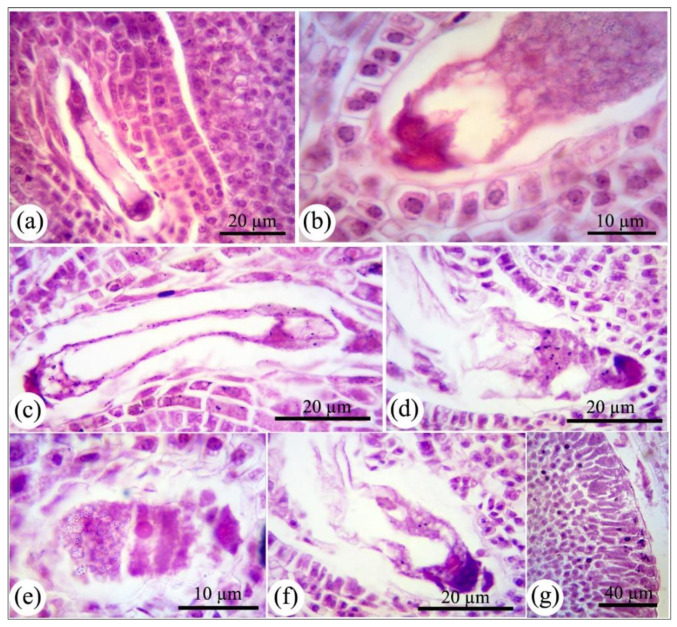
The development of female gametophyte in *Phaseolus vulgaris* in normal conditions (**a**,**b**) and treated with nCeO_2_ (**c**–**g**). Accumulation of starch granules inside the embryo sac and maternal tissues is observed after exposure to nCeO_2_ (**c**,**g**). Apoptosis and fragmentation in nuclei and cytoplasm are also considered important changes in female gametophytes after exposure to nCeO_2_ (**d**–**f**).

Male and female development preceding fertilization is divided into two main successive periods: sporogenesis (i.e., meiosis I and II in pollen mother cell (PMC) and megaspore mother cell) and gametogenesis (pollen and embryo sac development). The selected multistep photographs of the consecutive processes of male and female development in bean plants subjected aerially to nCeO_2_ compared to control plants are shown in Figure 1, Figure 2, Figure 3 and Figure 4, respectively.

### 2.1. nCeO_2_ Induces Alternations in Male Sporogenesis and Pollen Development

To analyze the effect of nCeO_2_ on the reproductive stages of bean plants (as a model for crop plants), we skipped the descriptive scenario of all stages preceding the reproductive process. Therefore, the occurred distinctive alternations within nCeO_2_ treatments compared to the control plant were only accounted to display in Figures. Our results did not show any abnormalities in flowering time and structural modification in floral organs between treatments and control plants. However, Du et al. [13] reported a one-week delay in the flowering of wheat plants grown in a field lysimeter exposed to 100 and 400 mg kg^−1^ nCeO_2_. This difference might be due to various time exposures, application methods and species type as well, as we exposed bean plants aerially to nCeO_2_ for two weeks while the wheat plants were grown in soil mixed with nCeO_2_ for about seven months in a trial done by Du et al. [13].

In general, pollen development in control plants initiated with the differentiation of pre-meiotic sporogenous mass within each anther locus. The differentiation of meiocytes was followed by the formation of a callose wall surrounding each of them. Tetrads of haploid microspores were produced after two consecutive meiotic divisions. Followed by the breakdown of the callose layer around tetrads, young microspores were released in the anther area and progressed to the late stage (i.e., mature pollen with completed wall and consisting of vegetative and generative cells). Monitoring sectioned samples of the consecutive stages of pollen development in all concentrations showed that only 2000 and partly 1000 mg L^−1^ nCeO_2_ concentrations caused traceable changes in microsporogenesis and pollen grains development. The male cells of nCeO_2_-treated plants normally developed as control male cells did until meiosis. During the sporogenesis period, the meiosis process (particularly at the chromosome pairing stage) was affected in the pollen mother cells at the highest concentration on nCeO_2_. As shown in Figure 1b, chromosomes have not been correctly separated during anaphase I (Figure 1a belongs to the control plant). Additionally, parts of chromosomes known as laggard chromosomes have been left in the cell center with no connection to the spindle apparatus. The statistical measurements from approximately 125 pollen mother cells (PMCs) per each treatment showed that the laggard chromosome was the most frequent abnormality observed at plants’ exposure to nCeO_2_ (1.14% and 2.48% in 1000 and 2000 mg L^−1^ nCeO_2_, respectively compared to control). Microsporogenesis analysis undertook abiotic stress conditions such as drought have shown a correlation between chromosomal behaviors during meiosis and plant fertility. It has been proven that the earliest stress-induced developmental defects such as chromosomal separation occur during meiosis, leading to unbalanced chromosome separation and then pollen sterility [20,21,22,23].

Followed by the pollen development progression, tetrad formation was also influenced by higher dosages of nCeO_2_, as the arrangement of microspores in tetrads was abnormal resulted in flattened and collapsed microspores. Further, some of the microspores have degenerated. They had smaller sizes than tetrads produced in control plants (Figure 1d in 2000 mg L^−1^ nCeO_2_ compared to 1b in control), similar to the abnormal microspores observed in bean plants under heat stress [7]. Moreover, microspores after release from the tetrads and their surrounded callose displayed various sizes ranging from relatively small to large in which were mostly wrinkle and amorphous (Figure 1f in 2000 mg L^−1^ nCeO_2_ compared to Figure 1e in control). Misshapen, collapsed, and probably non-viable pollens in nCeO_2_ treatments (more remarkably in 2000 mg L^−1^) were also demonstrated by SEM images (Figure 2f). Statistical analysis revealed a 47.89% abnormality (in terms of size, morphology, and being healthy) in pollen grains at the highest concentration of nCeO_2_, which was significant compared to control (26.62%) at *p* < 0.01 level. However, pollens showed no visible outward differences in other dosages.

Pollen abnormalities were also associated with starch accumulation in the highest concentration (Figure 2b), suggesting its distribution was more evident than control (Figure 2a). The starch accumulation could be due to the down-regulation of starch catabolism enzymes such as beta-amylase identified in our previous study [2], or inhibition of sugar utilization. The disturbance in carbohydrate metabolism is strongly related to abiotic stresses and may be involved in pollen development [21,24]. Indeed, the inhibition of starch catabolism into sugar, which protects membrane integrity, might affect pollen viability [20]. It is worth mentioning that pollen grains exposed to nCeO_2_ at the highest concentration showed cytoplasmic material exudation through colporates (Figure 2d), representing the cell wall and membrane damage resulting in losing the integrity and stability of pollen grains (Figure 2b). This phenomenon resulted in collapsing a large proportion of mature pollen grains together (Figure 3b). The total wall thickness of pollen grains also seems to be thinner in 2000 mg L^−1^ nCeO_2_ (Figure 2b) compared to control (Figure 2a). Additionally, the 2000 mg L^−1^ nCeO_2_-treated plant pollen cytoplasm appeared to be disturbed (Figure 2b,d). These findings suggest that nCeO_2_ at higher concentrations could have a serious effect on pollen wall architecture. Here, the change in wall conformation in pollen grains is also in agreement with increased membrane damage and electrolyte leakage of the same plant leaves subjected to nCeO_2_ during earlier growth [2]. Lipid degradation could likely be a reason for the lack of membrane integrity in pollen grains, as evidenced by the increase of lipid peroxidation and membrane lipids degradation such as phospholipids and their polar heads, mono- and di-glycerides, and sterols [1,2].

### 2.2. nCeO_2_ at Higher Concentration Declines Pollen Viability

Pollen viability is the ability of pollen to affect fertilization and consequent development of fruit and seed. Here, pollen viability was analyzed using Alexander and Acetocarmen protocols to assay pollens’ functional performance under nCeO_2_ exposure. There was no significant change in pollen viability between control and the lowest concentration of nCeO_2_ (Figure 3f). Nevertheless, a dose-depended decrease was registered for the higher concentrations as compared to control. The statistical analysis showed that 84.94% of tested pollens of control plants viable, while it declined to 67.89% and 58.80% at the 1000 and 2000 mg L^−1^ nCeO_2_ treatments (Figure 3e). Acetocarmine-stained pollens particularly, in 2000 mg L^−1^ nCeO_2_, displayed crumpled and collapsed shapes and often plasmolyzed cytoplasm (Figure 3b). There was an interesting correlation between pollen viability with pollen morphology and total normality. As the pollen abnormality increased, the pollen viability decreased, especially at the highest concentration, as shown in graphs (Figure 3e,f) and acetocarmine staining images (Figure 3b). In agreement with these findings, it is worth mentioning that pollen grains stained with eosin-hematoxylin were also showed visible differences between control and treatments. Unlike control pollen grains, which exhibited strong staining (Figure 1e and Figure 2a), the treated pollen grains were not deeply stained (Figure 1f and Figure 2b), which indicated less viability. Most of the pollen grains in control and 250–500 mg L^−1^ treatments were observed with pollen tubes germinating (Figure 2g). However, at a higher dosage (2000 mg L^−1^), most of the pollens germinated with shorter pollen tubes than control (Figure 2h,i), indicating that they were non-viable consistent with pollen viability results. Disturbance in starch metabolism, which acts as a fuel for pollen tube growth, might reduce pollen germination and longitudinal growth [24,25]. As shown previously, the pollen grains of the highest concentration accumulated remarkable starch granules compared to control (Figure 2b). All these changes mentioned earlier could alter male fitness by declining the viable amount of pollens [26]. The main reason for reducing pollen viability could be forming dysfunctional pollens resulting from abnormalities such as imperfect chromosome separation during microsporogenesis and subsequent changes in microgametogenesis mentioned earlier. A high amount of nCeO_2_ caused a reduction in the relative water content of bean leaves during vegetative growth [2], which could indirectly result in water deficit of generative organs and disturbances in the water-carbohydrate soluble level [27], suggest the defective development of pollen cell wall and as a result non-functional pollen grains.

Nevertheless, the application of nCeO_2_ at lower dosages (particularly at 250 mg L^−1^) did not negatively affect pollen morphology. Even the percentage of healthy pollens in this treatment was higher than the control plants (Figure 3e). Accordingly, pollen viability was also higher (Figure 3f), suggesting that a low amount of nCeO_2_ might improve plants’ physiological status at early growth and development [2,3,28], which is critical for the proper development of reproductive components.

### 2.3. The Effect of nCeO_2_ on Female Cells Development

The ovule development in all nCeO_2_ treatments was initiated similar to that of control. Briefly, functional megasporocyte was normally produced from primordial cells, and following that, proper meiosis I and II led to producing megaspores (Figures are not shown). By progressing the embryo sac development, apoptosis’s visible symptoms were appeared in the nuclei at two-nucleate megagametophyte (Figure 4c) compared to control (Figure 4a). As seen in histological sections, apoptotic bodies of nuclei have been dissipated inside the embryo sac area. Apoptosis has been associated with the defense responses to abiotic stresses such as water deficiency, temperature, and UV radiation. ROS involvement in apoptosis has been demonstrated in the literature [29]. In our previous experiment, we reported the reduced antioxidant capacity that could be a clue of the increased ROS levels and probably the provoked oxidative stress in bean plants aerially exposed to nCeO_2_, and thus, leading to a redox balance disturbance [2,3]. Antioxidative activity depletion is considered a central event that triggers plant cell death pathways [30]. Therefore, the induced apoptosis in the embryo sac’s cellular components can be assigned as ROS-derived programmed cell death (PCD) under unfavorable conditions. Histological examinations and the serial analysis of ovaries showed that the nCeO_2_ stressed-ovules accumulated numerous black granules in embryo sac cytoplasm, nucleus, and integuments cells in plants exposed to 1000–2000 mg L^−1^ nCeO_2_ (Figure 4g), which likely are granules enrich in starch or amyloplasts [31]. These findings indicated the distribution of enzymes involved in starch metabolism at higher concentrations. In our previous work, proteomics analysis of the leaves exposed to nCeO_2_ showed the down-regulation of beta-amylase activity at 250 and 1000 mg L^−1^ [2]. Therefore, it could be suggested that this enzyme’s lower activity has caused the inhibition of starch catabolism and resulted in the accumulation of high levels of starch granules in the cells. Structural changes, including shrinkage and fragmentation of cytoplasm mass, were also observed in some of the embryo sacs leading to loss of integrity and forming a discrete cytoplasm (Figure 4e). Our results would confirm that the early development of the embryo sac is one of the most vulnerable stages of stressful circumstances.

An adequate supply of photosynthetic products is essential for competent plant reproduction. High dosages of nCeO_2_ have reduced the Chl content and down-regulated photosynthetic proteins [2,13], leading to a reduction in the nutrient supply for reproductive cells [25]. The insufficient supply can be presumed to be responsible for anomalies in developing female cells and embryo abortion. Considering that plants use various mechanisms to optimize plant fitness under stressful conditions, our experiment revealed that bean plants exposed to higher dosages adjust the expenditure of maternal resources during flowering by minimizing the female gametophyte development. This hypothesize could be a strategy to conserve resources to optimize plants’ acclimation to oxidative stress made by extra nCeO_2_.

### 2.4. Impact of nCeO_2_ on Seed-Related Parameters 

As abovementioned, we analyzed the effects of different concentrations of nCeO_2_ on sporogenesis, pollen, and embryo sac development. The pollen viability was also considered a vital prerequisite for competent fertilization. The processes taking place during the pollen development are determinant for final production. On the male side, environmental stresses could affect the quality and quantity of pollens, which are critical in germinating and the rate of pollen tube growth. These changes could severely affect the reproductive output, causing low seed and fruit sets [26,32,33]. As fruit set and seed set form the most authentic test for pollen viability, measuring these yield characteristics could confirm the pollen performance. Therefore, to investigate the final yield (i.e., seed production that is the result of proper cooperation of sporogenesis and gametogenesis in both male and female gametophyte development), several seed-related parameters, including the number of pods, the number of seeds per pod, and plant and seed dry weight were measured (Table 1). The bean seeds inside the sheath and those dried of control and nCeO_2_-treated plants are shown in the supplementary file (Figure S2). Limited experiments across a life cycle field study have indicated that nCeO_2_ affects the vegetative phase by altering the plant performance during the generative phase. Du et al. [13] reported that wheat plants exposed to 100 mg kg^−1^ nCeO_2_ produced embryos with larger vacuoles, whereas 400 mg kg^−1^ concentration caused a reduction in the vacuole numbers. The same experiment, however, showed no effects of nCeO_2_ on the final biomass and yield. In another study, the grain yield of wheat plants (*Triticum aestivum* L.) grown in greenhouse conditions improved after exposure to 500 mg kg^−1^ nCeO_2_ [34]. Among the measured parameters, the number of pods per plant was significantly affected by nCeO_2_, especially at the highest concentration, which recorded a 13.7% reduction compared to control plants. However, there was no significant difference between other concentrations with control. The seed number either per pod or per plant did not show significant differences. It was decreased by 13% and 18% per pod and plant at the highest level, compared to control. However, nCeO_2_ significantly altered the seed weight (*p* ≤ 0.05) as the lowest weight was recorded at 2000 mg L^−1^ (Table 1). The toxicity of the higher concentrations of nCeO_2_ was reflected in seed appearance as a higher rate of seeds had not completed their development and were shrinkage (Supplementary file, Figure S2). Grain filling is closely linked to the whole-plant status and its reserve resources [35,36]. We have identified several down-regulated proteins involved in photosynthesis (such as ribulose bisphosphate carboxylase small chain) during the vegetative growth of bean plants subjected to nCeO_2_ could induce early senescence and reduce the grain-filling periods [2]. They concluded that a decrease in storage recourse due to photosynthesis disturbances could be a reason for producing immature and non-filling seeds at the highest concentration of nCeO_2_. However, we cannot exclude the role of reduced relative water content in the grain filling rate, which was also reported in several studies [25,35,36]. In contrast with the mentioned findings, our results showed that a lower concentration of nCeO_2_ (250 mg L^−1^) improves the yield as the higher value was recorded for seed number and weight (Table 1). Moreover, we collected healthier seeds from this treatment (Supplementary file, Figure S2). The positive effect of nCeO_2_ at optimum dosages was reported in previous studies [2,3,37].

Taking all these effects together with histological findings, it seems that the higher frequency of chromosomal abnormalities might interfere with the proper tetrad formation during sporogenesis, which could be a reason for reduced pollen viability and subsequent consequences such as the decrease in seed yield. Here, 2000 mg L^−1^ nCeO_2_ treated plants showed lower seed productivity, which could be attributed to severe abnormalities observed in either pollen or ovule development. Therefore, based on the current undertaken experiment, it can be probably concluded that nCeO_2_ application at higher dosages has mutagenic behavior causing chromosomal aberration and apoptosis. Moreover, according to the earlier discussion and obtained data from seed productivity, it can be extracted that physiological and functional abnormalities that occurred during either vegetative or generative phases potentially caused severe impacts on the development of male and female gametes, indicating that the success of fertilization has likely been affected by the high amount of nCeO_2_.

### 2.5. Accumulation of Cerium in Seeds

**Figure 5:**
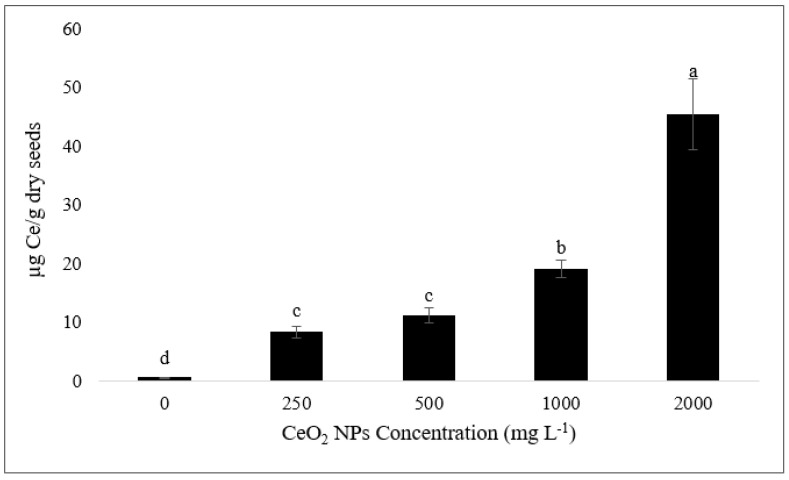
Cerium concentration in seeds harvested from bean plants treated aerially with different nCeO_2_ suspensions compared to control. Data are means ± SE. Different letters represent statistical differences at *p* ≤ 0.05.

Concentrations of cerium element (Ce) in bean plant leaves of this experiment were previously reported and used in this study to support the evaluation of Ce translocation. The calculated range of Ce content was recorded from 0.95 to 2.19 mg kg^−1^ [2]. In the current experiment, the ICP-MS assays showed the presence of Ce in dried seeds through a dose-dependent trend (Figure 5). As shown in Figure 5, the amount of Ce accumulated in seeds at the highest concentration (2000 mg L^−1^) was 45.4 µg per each kg^−1^ dried seed, showed a 98.7, 81.6, 75.4, and 58.1% increase compared to control, 250, 500, and 1000 mg L^−1^-treated plants, respectively. These data confirm the possibility of Ce translocation from leaves to seeds even though the translocation rate was very low. Previous studies have clearly shown the translocation of Ce from soil and root to up-ground parts of the plant (stem, leaf, and fruit) [2,37,38,39,40]. Our experiment indicates that even when nCeO_2_ is directly applied to leaves, these NPs can move throughout the leaf, translocate to the floral organs through the vascular bundle, and accumulate in the seeds. Recent investigations have shown that nCeO_2_-treated plants store most of the Ce accumulated in tissues as nCeO_2_ form, and only a small amount will be transformed to Ce ions [37,41]. Therefore, their findings and our results suggest that mature bean seeds may contain a considerable amount of nCeO_2_, which can be a serious warning for the human diet and health.

### 2.6. The Protein Content and SDS-PAGE Pattern of Seeds under nCeO_2_ Exposure

**Figure 6:**
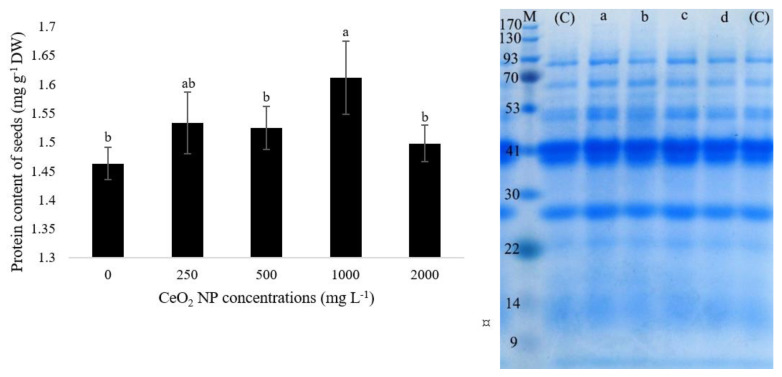
Total soluble protein content in bean seeds of plants exposed to different concentrations of nCeO_2_ compared to control (**left**). SDS-PAGE profile of protein extracted from bean seeds in control and treated plants (**right**). (C, a, b, c, and d are related to control, 250, 500, 1000, and 2000 mg L^−1^ concentrations, respectively). M: molecular mass marker (CinnaGen Company). Data are means ± SE. Means followed by the same letter are not significantly different (Duncan’s test *p* ≤ 0.05).

The total soluble protein content in the dried seeds was measured using the Bradford method. nCeO_2_ exposure significantly (*p* ≤ 0.024) affects the protein content in a non-linear trend (Figure 6). The seeds of plants exposed to all concentrations had higher protein content than control (Figure 6). The quantity was significantly higher at 1000 mg L^−1^ nCeO_2_ concentration, as it reached 1.61 mg g^−1^ DW (a 9% increase compared to control). The expression of new stress-related proteins could be a reason for this increase to counteract the detrimental effects of higher dosages of nCeO_2_. Our previous study identified several related-stress proteins, such as wound-induced basic protein and peroxidase, which were up-regulated upon exposure to 1000 mg L^−1^ concentration [2]. Indeed, in the same experiment, the proline content was also increased in nCeO_2_-treated plants. Overexpression of proteins involved in abiotic stress responses such as drought, salinity, heavy metals, and recently NPs have also been reported [38,42,43,44,45].

We performed SDS-PAGE electrophoresis on the extracted total protein of treated and control seeds to get more information. As shown in Figure 6, no visual changes were observed in the appearance and disappearance of protein bands between control and treatments. However, it is visualized that most of the protein bands increased their expression, especially at 250 and 1000 mg L^−1^ concentrations, which corroborates with the increase in protein content compared to control. These results suggest that nCeO_2_-treated plants have probably been counteracted with stress conditions only by changing the protein expression ratio. Therefore, there had been no need for the synthesis of new proteins. Considering the protein content results, slight visible changes in protein profiles were expected. Therefore, powerful and precise tools are needed to monitor the regulation of proteins. We suggest that further experiments on omics platforms such as proteomics to identify expressed proteins, particularly in bean seeds, to better uncover the molecular mechanisms.

## 3. Materials and Methods

### 3.1. Martials

The nCeO_2_ was purchased from Nanosany Company (US-Nano), Mashhad, Iran. As previously reported [2,3], the particles were spherical-shaped, measuring 10–30 nm with a 99.97% purity, a surface area of 30–50 m^2^ g^−1^, and 7.132 g/cm^3^ true density. The nCeO_2_ properties, including TEM and X-ray images, are presented in Supplementary materials. Other materials used in this study, including solvents, stains, and reagents, were all from Sigma-Aldrich Chemicals (Germany).

Seeds of *Phaseolus vulgaris* (var. pinto bean, F-16 commercial) were provided by Behineh Sazane Sabze Mehregan Company (biotech seed, Tehran, Iran). The used soil in this study was collected from a garden located in the greenhouse (Bu Ali Sina University, Hamadan, Iran; 34.7922° N, 48.4884° E). The soil was composed of 20% clay, 10.6% silt, 69.4% sand, and 2.3% organic matter. The pH was 7.6. 

### 3.2. Plant Growth Condition and CeO_2_ NPs Exposure Scenario

The bean seeds were sterilized by 5% sodium hypochlorite and germinated in moist sand. They were then planted in plastic pots (25 × 30 cm) containing three kilograms of the sieved garden soil and then incubated in greenhouse conditions. A 14 h photoperiod, day/night temperature average of 30 ± 2/16 ± 2 °C, and 70% humidity were steadily kept during the entire experiment. The thinning process was done after three weeks of growth, and plants with similar morphological features (almost in BBCH 13–15—phenological stage: 3–5 true leaves) were selected for the nCeO_2_ exposure scenario. The plants were regularly watered every 48 h during the entire growth cycle. No fertilizer and pesticide were used.

nCeO_2_ was suspended in Millipore water (MPW) to achieve the desired final concentrations (250, 500, 1000, and 2000 mg L^−1^) and subjected to 45 min sonic bath (Bandelin Sonorex, Faraz Tab Tajhiz, Iran) to ensure the full homogeneity of the nCeO_2_. The pH of nCeO_2_ was found to be around 6.5–7. The exposure dosages for nCeO_2_ were selected based on previously tested ranges in the literature, showing either positive or negative effects [2,46]. The pots containing three-week-old plants were then divided into five groups (four concentrations and one control) and five replicates per each. The leaves of bean plants were exposed to the aforementioned dosages of nCeO_2_ by regularly spraying every 48 h for two weeks. The volume of sprayed solutions was enough to wet the leaf area. Overall, a total volume of 200 mL of each concentration was applied. The soil surface was covered by thin aluminum foil to prevent contamination with NPs. Plants sprayed with MPW only, the same way as nCeO_2_, served as the control. After treatment, the pots were kept in the greenhouse in a stable condition until full maturity. The sample harvesting was done several times from the beginning of flowering. Different sizes of flowers ranged from closed buds to fully open flowers were collected from each treatment. At the end of the growth cycle, the pods containing seeds were also collected. The seeds were collected from pods. The weight (g) of 10 bean seeds was noted per each replicate. The total number of seeds per pod and plant was also recorded and stored at 4 °C for total protein and SDS-PAGE analyses.

### 3.3. Sample Preparation for Histological and Microscopy Analysis

On the day of flowering, flowers in different sizes from closed buds to fully opened ones were carefully dissected using a razor blade from each replicate per treatment. The samples were immediately rinsed using distilled water and fixed in FAA_70_ containing 3% formaldehyde, glacial acetic acid, and 70% ethanol at 2:1:17 proportions, respectively, for about 24 h. After this, the samples were rinsed with distilled water several times to remove the FAA solution residual and then stored in 70% ethanol at 4 °C for microscopy analyses. Before viewing the floral structures, the samples were dehydrated in a graded ethanol series ranging from 50–80% (each step one time) to 100% (two times) at room temperature. The samples were also exposed to toluene (pure) for 20 min two times to full dehydration. At the end of sample preparation, floral bodies were embedded into paraffin liquid and incubated in an oven at 70 °C for 2–3 days (this time depend on floral size). To prepare tissue sections, flat-thin ribbons were made using a microtome blade at 7-micron thickness (Micro DS 4055, Germany). Ribbons were drop-fixed in a 3% formalin solution and left to dry over a heater at 40 °C. The samples were stained using Haematoxylin-Eosin staining following a standard protocol [47]. A LABOMED model LX50 microscope (Germany) and LABOMED digital camera were used to view the dynamic changes. For scanning electron microscope (SEM) analysis, the critical-point dried pollen grains from dehisced anthers were mounted on aluminum stubs and coated with gold. The samples were analyzed using a JSM-840A, JEOL SEM (Tokyo, Japan). At least ten samples of each developmental stage per each pot and over 100 experimental slides were monitored to investigate the possible changes.

### 3.4. Pollen Viability Test

Two different staining methods were assessed to test pollen viability: (1) Acetocarmine and (2) Alexander. Briefly, in the Acetocarmine method, dried pollen grains were spread on a glass microscope slide, and then a drop of 1% Acetocarmine was trickled on it. After that, the slides were carefully heated at 30 °C for 10 min. In this technique, the pollen grains were considered viable if they turned red, but non-viable pollen grains showed a gradation in color from very light pink to colorless. The Alexander solution was made by the compounding of ethanol 96%, 10 mL; 1% malachite green in 95% ethanol, 1 mL; distilled water, 50 mL; glycerol 25 mL; phenol, 5 gm; chloral hydrate, 5 gm; acid fuchsin 1% in water, 5 mL; orange G, 1% in water 0.5 mL; and 4% glacial acetic acid, 1–4 mL. The anthers were immersed into an adequate aforementioned prepared solution for 3 h at 60 °C. In this method, purple or pink color indicated viable pollen while the pale one non-viable. The figures were captured using a LABOMED digital camera (Medac, Wedel, Germany). At least 20 slides were screened for evaluation of the pollen grain viability in both techniques. Finally, pollen viability was determined by counting viable pollens out of at least 50 pollen grains per prepared slide (20 slides per treatment).

### 3.5. Determination of Ce Content in Tissues

The dried seeds were washed by agitation with MilliQ water for 30 min to remove any possible contaminations. An accurate aliquot (0.5 g) of dried seed was powdered and digested using 6 mL of HNO_3_ 65%, 2 mL of H_2_O_2_, and 0.1 mL of HF 48% in a Teflon pressure vessel in a microwave oven (ETHOS One; Milestone Inc., Sorisole, Bergamo, Italy). After mineralization, the digested extract was filtered through a 0.45 µm filter, diluted to 50 mL with MilliQ water, and analyzed for Ce ion content by an Inductively Coupled Plasma-Mass Spectrometry (ICP-MS) with an octopole reaction system. (Agilent7900, Agilent Technologies, Santa Clara, CA, USA). The accuracy of the analytical procedure was inspected using a standard running solution.

### 3.6. Determination of Total Protein and SDS-PAGE Analysis

To extract total protein, dried seeds were grounded in 3 mL extraction buffer (25 mM KH_2_PO_4_ at pH 7.4) in liquid nitrogen and centrifuged at 10,000× *g* for 15 min. The Bradford assay was used to determine protein concentration at 595 nm. SDS-polyacrylamide gel electrophoresis (SDS-PAGE) was performed following a standard protocol [48]. Briefly, the protein extract was mixed with loading buffer equally, and then 20 µL of the mixed sample was run into a 12% polyacrylamide gel at a constant voltage (90 V). Coomasie Brilliant Blue R-250 was used to stain the gel. Molecular weight standards containing ten bands were purchased from CinnaGen, Iran. 

### 3.7. Statistical Analysis

PASW Statistics 18.0 (SPSS 19.0 package, Chicago, IL, USA) was used to analysis of variance (ANOVA; *p* < 0.05) in quantitative values of cerium content, pollen irregularity, protein content, seed weight, and seed yield. The statistical differences between each treatment data were processed by post hoc test on a mean basis. All experiments were performed in five replicates.

## 4. Conclusions

The current study was undertaken to fill the existing gaps in the possible changes in generative and reproductive stages of plant growth after exposure to nCeO_2_. The finding confirmed that, besides the well-documented effect of nCeO_2_ on the physiological, biochemical and molecular status of the vegetative phase of plant growth [2,3], subsequent plant development processes such as reproductive periods are known to be vulnerable to the environmental condition would be affected. The results have shown that nCeO_2_ displays toxicity to reproductive processes, especially pollen fitness, and yields productivity at the tested dosages except for the lowest concentration.

Based on the antecedent of plants grown under different dosages of nCeO_2_ showed various alternations in the morphological, metabolome, and proteome profiles [2], the findings derived from the present study suggest that the vegetative plant growth status might mediate possible responses of the reproductive cells to nCeO_2_ treatment. According to the connection between our previous results obtained from vegetative growth of bean plants at the molecular levels, it can be concluded that misfolding of proteins, changes in antioxidative metabolites, hormonal imbalance, and increased ROS could be possible reasons for defects in developmental processes (i.e., male and female development) and eventually lower yield in plants exposed to higher dosages of nCeO_2_. Overall, we suggest that the bean plants’ yield is likely dependent on the physiological status prior to flower formation. Our results draw attention to the fact that, although nCeO_2_ itself likely has less influence on the structure and function of the female development than on the male, it may still cause severe yield losses when associated with water deficiency resulting from increased electrolyte leakage at higher dosages [2].

## Figures and Tables

**Table 1:** The number of pods per plant, seeds per pod, seeds per plant, and seed dry weight in seeds harvested from control and nCeO_2_-treated plants. Values are expressed as mean ± SE. Different letters represent a significant difference between concentrations.

nCeO_2_ Concentrations (mg L^−1^)	0	250	500	1000	2000
Number of pods per plant	13.3 ± 1.63 ab	14.1 ± 2.46 a	13.1 ± 2.02 ab	13.8 ± 1.31 a	11.7 ± 1.15 b
Number of seeds per pod	5.2 ± 1.03 ab	5.8 ± 1.13 a	5.1 ± 0.99 ab	5.1 ± 1.10 ab	4.6 ± 0.69 b
Number of seeds per plant	67 ± 6.2 ab	68.3 ± 4.2 a	66 ± 6.5 ab	67.6 ± 7.3 ab	54.6 ± 9.1 b
Seed dry weight (g/10 seeds)	3.19 ± 0.19 a	3.31 ± 0.21 a	3.23 ± 0.21 a	3.11 ± 0.21 a	2.6 ± 0.29 b

## Supplementary Materials

The following are available online at https://www.mdpi.com/article/10.3390/nano11040862/s1, Figure S1: TEM (left) and X-ray (right) images of nCeO_2_ for particle characterization of size and morphology. Figure S2: The seeds of control (a and d) and nCeO_2_-exposed plants (b, d, and e images are related to 250 and 1000 mg L^−1^ concentrations).
