# Supplementary material for: Foliar Application of CeO2 Nanoparticles Alters Generative Components Fitness and Seed Productivity in Bean Crop (Phaseolus vulgaris L.)
Source: Nanomaterials (Basel). 2021 Mar 28;11(4):862. doi: 10.3390/nano11040862 (PMC8065513; doi:10.3390/nano11040862)
Supplement: Supplementary file 1 [file nanomaterials-11-00862-s001.pdf]

nanomaterials

MDPI

Supplementary materials

Foliar Application of CeO_2 Nanoparticles Alters Generative Components Fitness and Seed Productivity in Bean Crop (*Phaseolus vulgaris* L.)

Hajar Salehi [1], Abdolkarim Chehregani Rad [1,*], Ali Raza [2] and Jen-Tsung Chen [3,*]

[1] Laboratory of Plant Cell Biology, Department of Biology, Bu Ali Sina University, Hamedan 65174, Iran; hajarsalehi@ymail.com
[2] Key Lab of Biology and Genetic Improvement of Oil Crops, Oil Crops Research Institute, Chinese Academy of Agricultural Sciences (CAAS), Wuhan 430062, China; alirazamughal143@gmail.com
[3] Department of Life Sciences, National University of Kaohsiung, Kaohsiung 811, Taiwan
* Correspondence: chehregani@basu.ac.ir (A.C.R.); jentsung@nuk.edu.tw (J.-T.C.)

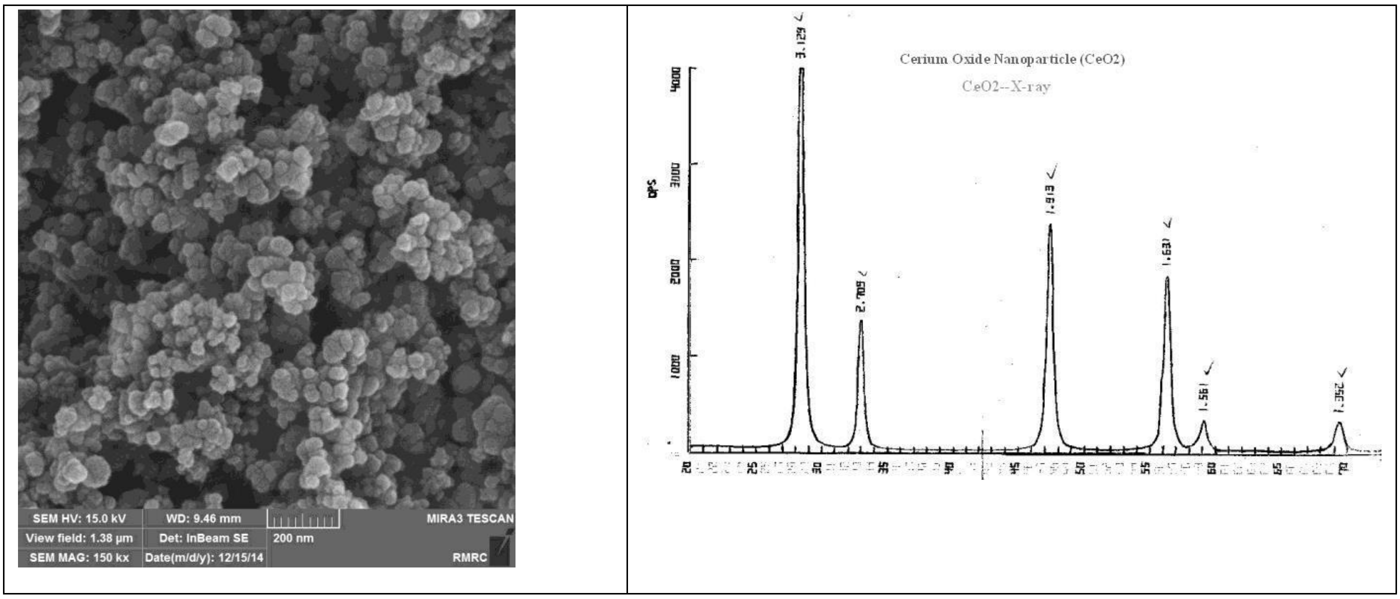

Figure S1. TEM (left) and X-ray (right) images of $nCeO_2$ for particle characterization of size and morphology.

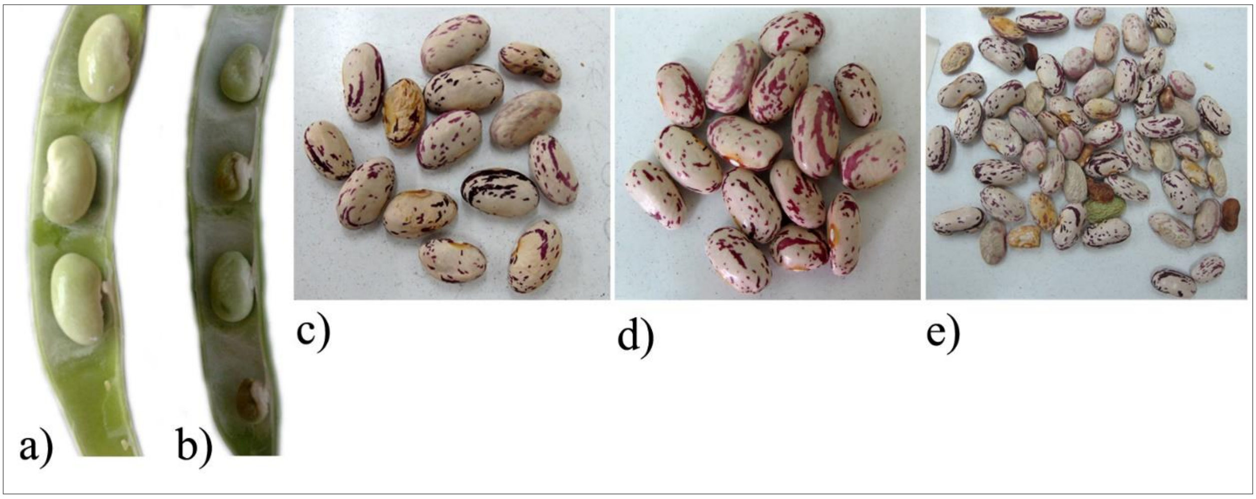

Figure S2. The seeds of control (a and d) and $nCeO_2$-exposed plants (b, d and e images are related to 250 and 1000 mg L^{-1} concentrations).
